# The use of preferred social stimuli as rewards for rhesus macaques in behavioural neuroscience

**DOI:** 10.1371/journal.pone.0178048

**Published:** 2017-05-25

**Authors:** Helen Gray, Bradley Pearce, Alexander Thiele, Candy Rowe

**Affiliations:** Centre for Behaviour and Evolution, Institute of Neuroscience, Newcastle University, Newcastle upon Tyne, United Kingdom; University of Verona, ITALY

## Abstract

Macaques are often motivated to perform in neuroscientific experiments by implementing fluid restriction protocols. Daily access to water is controlled and the monkeys are rewarded with droplets of fluid for performing correct trials in the laboratory. Although these protocols are widely used and highly effective, it is important from a 3Rs perspective to investigate refinements that may help to lessen the severity of the fluid restriction applied. We assessed the use of social stimuli (images of conspecifics) as rewards for four rhesus macaques performing simple cognitive tasks. We found that individual preferences for images of male faces, female perinea and control stimuli could be identified in each monkey. However, using preferred images did not translate into effective motivators on a trial-by-trial basis: animals preferred fluid rewards, even when fluid restriction was relaxed. There was no difference in the monkeys’ performance of a task when using greyscale versus colour images. Based on our findings, we cannot recommend the use of social stimuli, in this form, as a refinement to current fluid restriction protocols. We discuss the potential alternatives and possibilities for future research.

## Introduction

Macaques are used in neuroscientific experiments as models to better understand human brain function. This often requires their engagement in laboratory tasks, which can require many hundreds of trials per day to achieve adequate statistical power. In order to motivate the animals, many researchers reward them with highly-valued fluid upon the completion of each correct trial (e.g. [1,2]). Where studies require large numbers of trials, fluid restriction protocols, which control the daily access to fluid for an animal, may have to be employed to sufficiently motivate the monkeys to consistently perform the task (see [3] for a review). Fluid restriction is traditionally a controversial topic due to the potential negative impacts on the animals’ physiology, behaviour and welfare [4–6], although recent research suggests that many of these concerns are unfounded [7]. However, it is still important that alternatives to fluid rewards are explored for their efficacy and practicality in motivating macaques to perform adequate numbers of trials in the laboratory on a daily basis (referred to as ‘adequate task performance’ from here onwards). One such potential alternative is the use of social stimuli as rewards.

In the wild, rhesus macaques live in large social groups, where they play, have social contact and forage together [8,9]. In UK scientific holding facilities, extensive grouping is not always possible, and more typically, animals are housed in smaller social groups or in pairs. Sometimes, in exceptional circumstances, individuals may even have to be housed alone, such as when a monkey is in a pre- or post-operative state or has experienced a bout of aggression with a cage mate. Given that under these holding conditions animals have less social contact than their conspecifics in the wild, it is worth speculating that macaques’ natural sociality could be capitalised upon when exploring alternative rewards. Indeed, some of the early studies of social reward showed that rhesus macaques would perform behaviours, such as opening a window, in order to gain visual access to a conspecific [10]. Since then, numerous studies have shown that non-human primates (NHPs) will perform a wide range of tasks to gain social rewards, including: lever pressing [11,12], pushing on a panel [13] and manipulating a joystick [14]; for full review see [15].

In addition to this, preferences for viewing specific types of social rewards have also been reported. For example, both male and female rhesus macaques prefer viewing photographs of unfamiliar, rather than familiar, conspecifics [16,17], female stump-tailed macaques (*Macaca arctoides*) show more interest in photographs of infant stump-tailed macaques and females carrying infants compared with pictures of lone adult females [18], and female rhesus macaques choose to view faces of dominant male macaques but are less interested by low-status males [19]. This suggests that the social structure of macaques determines specific preferences in social stimuli and that images are viewed to gain social information.

However, whilst these kinds of social stimuli are clearly of interest to macaques, the question remains as to whether they are sufficiently motivating to function as a reward for adequate task performance. Some studies suggest that this may be the case, and argue that social stimuli provide a viable alternative or supplement to fluid or food rewards. In a task where male rhesus macaques chose between a smaller fluid reward coupled with a social image or a larger fluid reward with no image, males would sacrifice fluid to view images of dominant males and female perinea, but required an ‘overpayment’ of fluid to view subordinate males [20,21]. Other studies suggest that interest in viewing videos and photographs can be maintained by changing stimulus sets [17,22,23], indicating that novelty may be an important factor in prolonging interest. Although these studies demonstrate that social stimuli can be rewarding, some of the studies [22–24] were carried out with macaques that were individually housed, a factor which could potentially increase the reward value of these stimuli.

Taking into account the evidence surrounding the potential motivational value of social stimuli, this study investigates to what extent social stimuli can be used to motivate macaques in a behavioural neuroscience setting for adequate task performance, with the aims to reduce the need for fluid rewards, and refine protocols associated with fluid control. We tested both greyscale and colour images to explore whether they had different motivational values. Four male rhesus macaques involved in behavioural neuroscience studies (outside the context of the current study) were assessed for their individual preferences for social images and were then rewarded with a choice of fluid or an image from their preferred stimulus set for the completion of a simple cognitive task.

## Materials and methods

Four monkeys, weighing between 8–14.5 Kg at the start of the experiments, were used in this study. All experimental procedures complied with the European Union Directive 2010 (2010 / 63 / EU), the National Institutes of Health (Guidelines for Care and Use of Animals for Experimental Procedures), the Society for Neurosciences Policies on the Use of Animals and Humans in Neuroscience Research, and the UK Animals Scientific Procedures Act. The study was approved by the Animal Welfare Experimental Review Board (AWERB) of Newcastle University.

Subjects were each housed in a stable pairing with another male (not participating in this particular study), in a cage sized either 2.1 x 3.0 x 2.4 m or 2.3 x 2.45 x 2.4 m. The facility allowed individuals to have visual, olfactory and auditory contact with monkeys in adjacent cages. Toys were given on a rotational basis as an environmental enrichment, and dry food mix (Mazuri Primate Expanded, Old World Monkey Banana Chunks, Trio Munch Rings and LP Forage Mix, *Special Diet Services*; Monkey Diet, *LabDiet*^®^, *IPS Ltd*) was added to shavings that covered the floor to encourage foraging, as a stimulant and reward [25], as recommended by primate welfare guidelines [26]. Each cage had 3 wooden perches, a wooden shelf (running the width of the cage) and a ‘balcony’ located at the top of the cage as well as two fire hoses handing from the ceiling. The diet given was supplemented with fruit and vegetables once a week. When animals were not taking part in experiments, water was freely available in the home cage. The facility had a 12:12 light/dark cycle from 7 am to 7 pm, as well as natural light from ceiling windows. The temperature and humidity were approximately 20°C and 24%, respectively.

The experimental tasks required the macaques to be given controlled access to fluids. The fluid control protocol consisted of 5 days of fluid control (daily allowance given from Sunday to Thursday) with free access to water after completing work on Friday and all-day Saturday. The minimum daily fluid allowance was a volume of water that sufficiently motivated the monkey to perform in the laboratory task, and is expressed as a percentage of their consumption when given free access to water and by the ml/Kg/day (Monkey 1 = 25% or 13 ml/Kg/day; Monkey 2 = 53% or 25ml/Kg/day; Monkey 3 = 30% or 32 ml/Kg/day; Monkey 4 = 40% or 22 ml/Kg/day). Through their participation in experiments, individual monkeys could earn above their daily fluid allowance. However, this did not occur every day, and on days where monkeys did not reach their daily fluid allowance during the experimental task, they were supplemented with additional water to reach their daily allowance. Each monkey was taken to the laboratory at approximately the same time each day, with times differing between monkeys.

During the study, monkeys underwent daily checks by a technician or veterinarian, where fur condition, faeces, eyes, food intake and activity levels were visually assessed. In the event of any health or welfare concern, technicians and the veterinarian checked the animal several times per day. Monkeys were sedated annually to assess their general health.

### Images

Digital photographs (3072 x 4608 pixels, 24 bit colour depth) of adult rhesus macaques were taken at the German Primate Centre (DPZ) in Göttingen, Germany, in October 2013, using a Nikon 1 V2 camera. Photographs were taken of macaques in large, outdoor enclosures. Of the images taken, a subset of neutral faces from dominant males and of female perinea were selected to be edited in both greyscale and colour to produce the image sets described in the next section. The backgrounds of the photographs were removed to avoid any elements of interest detracting from the focal image.

#### Greyscale images

70 greyscale images were used in total (35 male and 35 female). Seven males were photographed, with 5 images used from each individual. Due to the difficulty in getting good quality images of female perinea, seven females were photographed, with 2–7 usable images for each individual.

**Fig 1:**
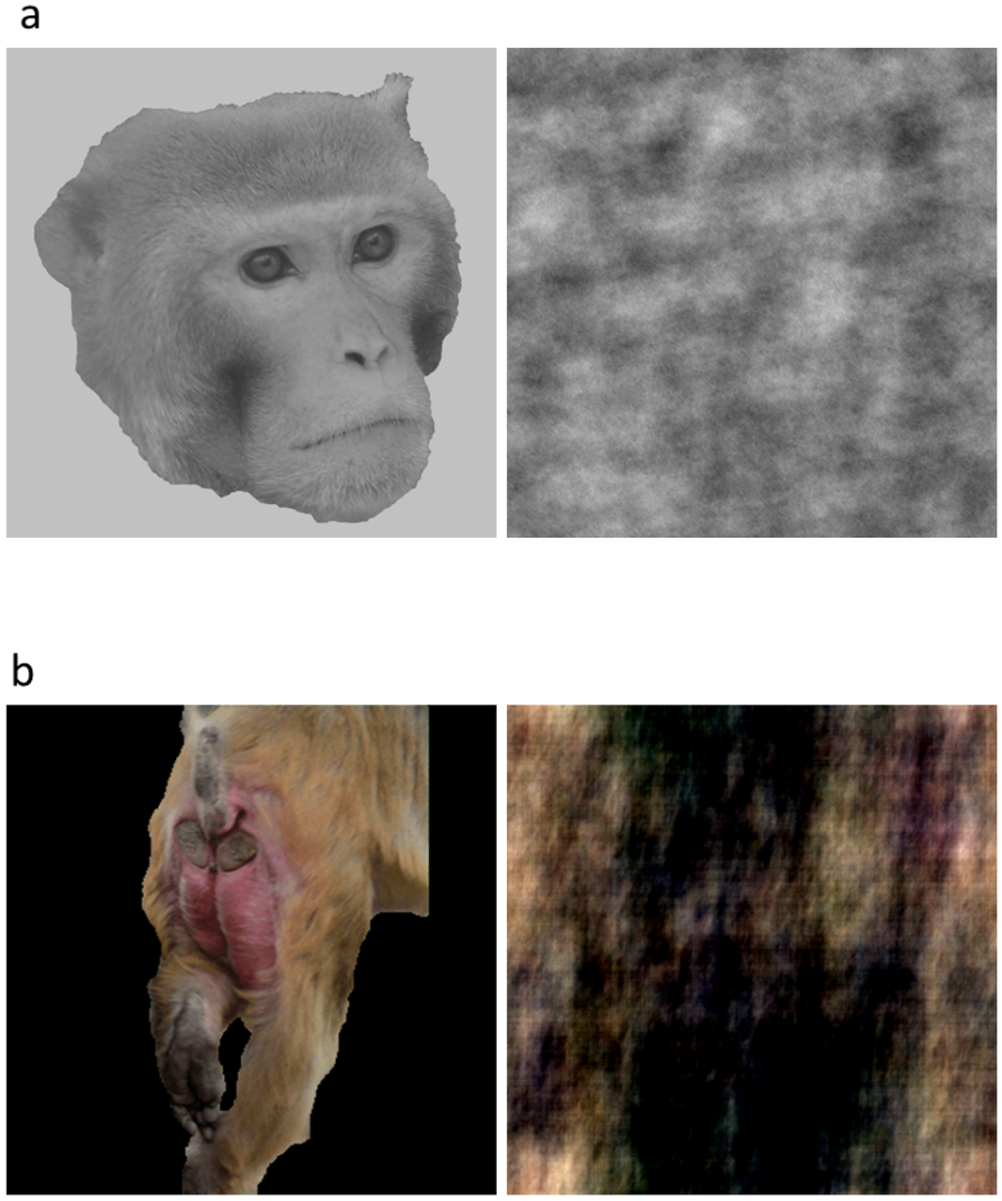
Examples of greyscale (a) and colour social images (b) and their matching control images (not to scale) which were 326 x 326 pixels and presented at 8.85 x 8.85 degrees of visual angle (dva).

The original photographs were converted to greyscale and were edited to normalise contrast and luminance across all photographs. Control images were produced by scrambling the facial and perineal images such that the distributions of contrast, luminance and spatial frequency were identical to the original image (Fig 1a). This was achieved by performing a Fourier transformation and calculating the amplitude and phase spectrum. A random phase structure was then generated and added to the phase spectrum of the Fourier transform. The amplitude spectrum was then combined with the phase spectrum and an inverse Fourier transformation was performed. Scrambled images were chosen over other types of control images, such as landscapes, because they retained the second order statistics of the original images, allowing us to test the efficacy of the social stimuli, without the complication of containing other potentially motivating/rewarding features in the control image. Our control images were therefore sufficiently neutral to test whether social rewards can be used as supplements or replacements of fluid control, whilst ensuring that other differences (e.g. second order image statistics) between social and control stimuli did not confound the interpretation of our results. All social images and control images were resized to 326 x 326 pixels.

#### Colour images

The same set of photographs used for the greyscale images was used for testing colour images. However, the contrast in one male’s images made it impossible to enable colour normalisation, and this individual was omitted from the colour image set, leaving 30 male images. Five images (the poorest in quality, e.g. more blurred) were removed from the female stimulus set to leave 30 images (six females with 2–7 images for each). 60 colour images in total were tested.

To normalise the colour images, chromaticity values of the images were converted to CIE colour space values. The colour space values were then converted to red, green, blue (RGB) values using the pre-defined matrix. Desired RGB values on the CRT screen were obtained by gamma correction of the monitor [27]. This ensured that images were presented in device independent Yxy chromaticity coordinates. The image backgrounds were removed and replaced by a homogenous grey with a luminance matching the mean luminance of the image pixels. The mean image luminance (CIE 1931 Y, 2 degrees observer) of each image was calculated based on the gamma correction values. The mean luminance of each image was then taken away from that image, so that its mean luminance was 0, with the standard deviation being the mean luminance contrast. The mean luminance of the complete set of images was added to each image, such that each image had the same mean luminance. Finally, CIE Yxy values were converted back to RGB using the inverse of the transform created when calibrating the screen. If pixel values fell outside of a displayable range, they were returned to their original values, which allowed for complete images whilst only slightly altering the mean luminance. The final mean luminance was calculated and the images were converted to Microsoft Indexed 255 Colour Bitmaps with dimensions of 326 x 326 pixels. Control images were scrambled images that were created in the same way as the greyscale images (Fig 1b).

### Image preference test

All stimuli were presented on a GDM F500R computer monitor, with an 85 Hz refresh rate and 1280 x 1024 pixel resolution. Stimulus presentation, reward delivery and experimental timing were controlled using Cortex (DOS-Version 5.95; IMH, http://dally.nimh.nih.gov/). Eye movements and position were monitored by infrared eye monitoring technique. The right eye was illuminated with an infrared light source and movements were monitored by the TREC ET-49 (v 1.2.8) software package, sampled at 240 Hz, and x- and y- eye position data were stored at 250 Hz each. To ensure accurate saccade tracking during testing, the subject’s eye movements were calibrated each day, prior to testing.

**Fig 2:**
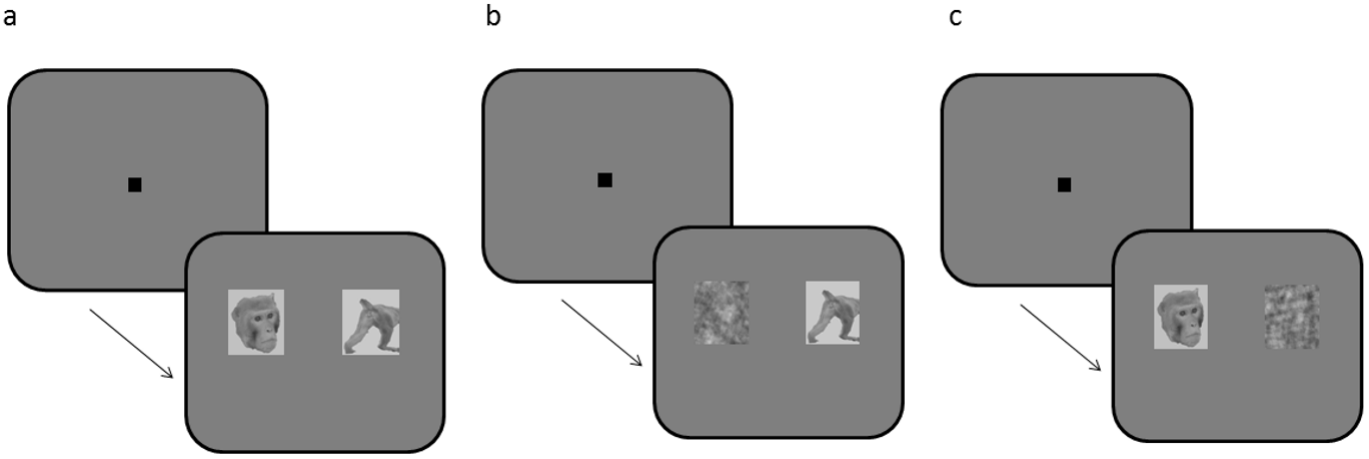
Social reward preference test. The monkey fixated on a central dot for 2000 ms before a pair of images was presented. The pairs could be either: male face vs female perinea (a), female perinea vs a matched control image (b) or a male face vs a matched control image (c). Image pairs were presented for 5000 ms and the monkey could look wherever he chose for this duration.

Monkeys 1 and 2 were tested with greyscale images and Monkeys 3 and 4 with colour images to assess whether one image type was more motivating than the other. During the experimental sessions the animals were head fixed (for surgical and experimental details see [28]). Each monkey had to fixate on a central spot (0.5 x 0.5 degrees of visual angle, dva; 5 x 5 dva eye window allowance) for 2000 ms, after which a juice reward of Ribena was given (~0.1–0.2 ml, dependent on the monkey). Following the initial 2000 ms of fixation, two images (8.85 x 8.85 dva) were presented for 5000 ms (Fig 2), during which the monkey was free to look wherever he chose on, or off, the screen. The monkey’s right eye was tracked throughout these 5000 ms to ascertain which image he spent more time looking at (if any). Trials were conducted in blocks; once an image had been used, it would not be shown again until all other images had been presented. Once all images had been used, a new block began. This ensured that each image was shown an equal number of times per session. The images were shown in three different pairings, as follows: male vs scrambled control, female vs scrambled control, and male vs female. The control images were shown alongside their matching original monkey image, whereas male-female image presentations were paired at random. Reward was given after successful fixation, instead of at the end of a trial (following the 5000 ms image presentation), to ensure that no association could be made between the monkey’s choice of which image to view and the fluid reinforcement.

This protocol was carried out for 4 days for each monkey. Following this, the eye tracking data were analysed and the image type (male face, female perinea or scrambled control) with the longest-associated average viewing time was taken as the monkey’s preference. The preferred image type was specific to each animal, and animals received their own preferred image type in the fluid + image reward task.

### Using the preferred images as rewards

**Fig 3:**
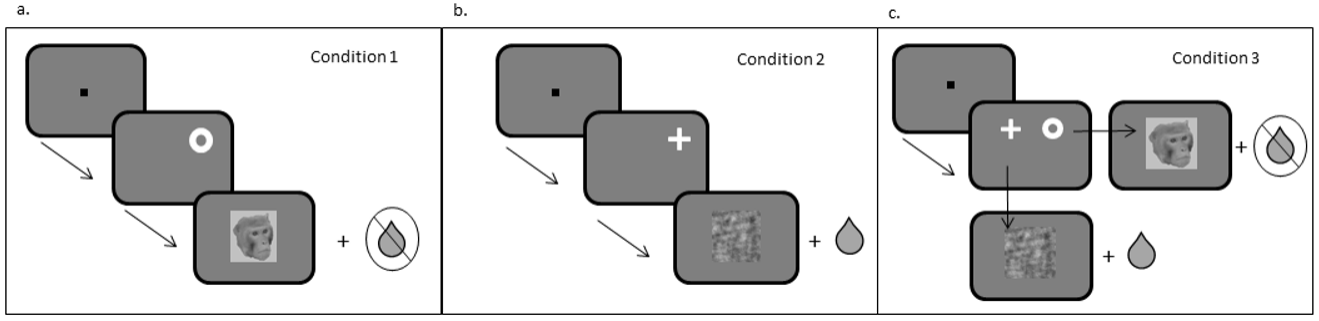
The three conditions of the fluid + image reward task. The monkeys completed either a bar release (Monkeys 1 and 2) or fixation (Monkeys 3 and 4) before being presented with one of three conditions. Condition 1 (a) and Condition 2 (b) represent learning trials. In Condition 1 a saccade is made to an annulus to receive a preferred stimulus with no fluid. In Condition 2 the monkeys saccade to a cross to receive a fluid reward and a non-preferred image. In Condition 3, the monkeys choose between their rewards.

Two slightly different tasks were used to test if preferred images were rewarding in cognitive tasks, depending on each monkey’s previous training. These are referred to as the fluid + image reward tasks. Monkeys 1 and 2 were required to hold a touch bar whilst fixating on a central square (0.5 x 0.5 dva; 5 x 5 dva eye window allowance) on the screen for 1650–2000 ms and release the bar within 1000 ms when the square dimmed (reduced in contrast). Monkeys 3 and 4 were required to fixate on a central square for 1000 ms with no bar release required. Although tasks differed between monkeys, the nature of the task was not important; only that a monkey could perform consistently. On completion of a correct bar release or fixation, the monkeys were confronted with one of three possible conditions (Fig 3). Either a single annulus (condition 1) or a single cross (condition 2) were presented randomly at either (x = -6, y = 6 dva) or (x = 6, y = 6 dva), or both stimuli were presented simultaneously (condition 3), whereby the location of the cross/annulus was assigned randomly to the two possible stimulus locations. To obtain a fluid reward (Ribena, ~0.1–0.2 ml, dependent on monkey) and the presentation of a non-preferred image stimulus (8.85 x 8.85 dva) for 2000 ms, the monkey had to make a saccade to the cross and fixate on it for 500 ms. To view an image from his preferred stimulus set (8.85 x 8.85 dva) for 2000 ms with no fluid reward, the monkey had to saccade to the annulus and fixate for 500 ms. If no saccade was made, a 2000 ms delay occurred before the next trial, without any reward (fluid or image) being given. Conditions 1 and 2 served as ‘learning trials’ to help the monkeys to establish the outcome of a saccade to each target. The choice trials (condition 3) established which the monkeys’ preferred reward (fluid or image) was. Monkeys 1 and 2 had received some prior training on the meaning of the saccade targets and so 20% of their daily trials were learning trials and 80% were choice trials. Monkeys 3 and 4 were not trained beforehand on the meaning of the targets (due to time constraints) and so had an equal proportion of all three trial conditions daily (66% were training trials to the annulus and the cross, and 33% were choice trials), to ensure that they learnt what the targets represented.

To measure the number of trials performed solely for fluid, monkeys performed a control task (hereafter referred to as the fluid only task). This consisted of the annulus saccade target and a resulting fluid reward with no image presentation, as well as a 2000 ms delay between trials to mimic the timings of the fluid + image reward task timings. The two tasks (the fluid + image reward task and the fluid only task) were carried out in an ABBABAAB order over 8 days, at the monkeys’ normal fluid allowance levels, to establish a baseline interest in the images.

### Statistical methods

All analyses were carried out using IBM Corp. SPSS (v21, SPSS Inc, Chicago, USA). Image preference was established by analysing the number of ms the monkey spent looking at each of the two presented stimuli per trial. These data were not normally distributed for any of the monkeys, could also not be transformed to normality and so were analysed using Wilcoxon Signed Rank tests.

The rewarding value of the social stimuli was investigated using *t* tests to compare the mean number of correct trials performed in the fluid + image reward task and the fluid-only task (the data were normally distributed and in cases where assumptions of equal variances were violated, *p*-values were adjusted as necessary). We also assessed preferences within the fluid + image reward task by calculating the percentage of times either fluid or social rewards were chosen in trials where the monkeys had a choice (omitting the ‘learning trials’). This allowed us to establish, in conditions when the monkeys were given a choice, whether they were motivated to work for fluid or image rewards. We conducted analyses separately for each monkey to assess individual results and, to increase statistical power, we then pooled the data from all monkeys and performed a repeated measures ANOVA.

## Results

### Image preference

**Fig 4:**
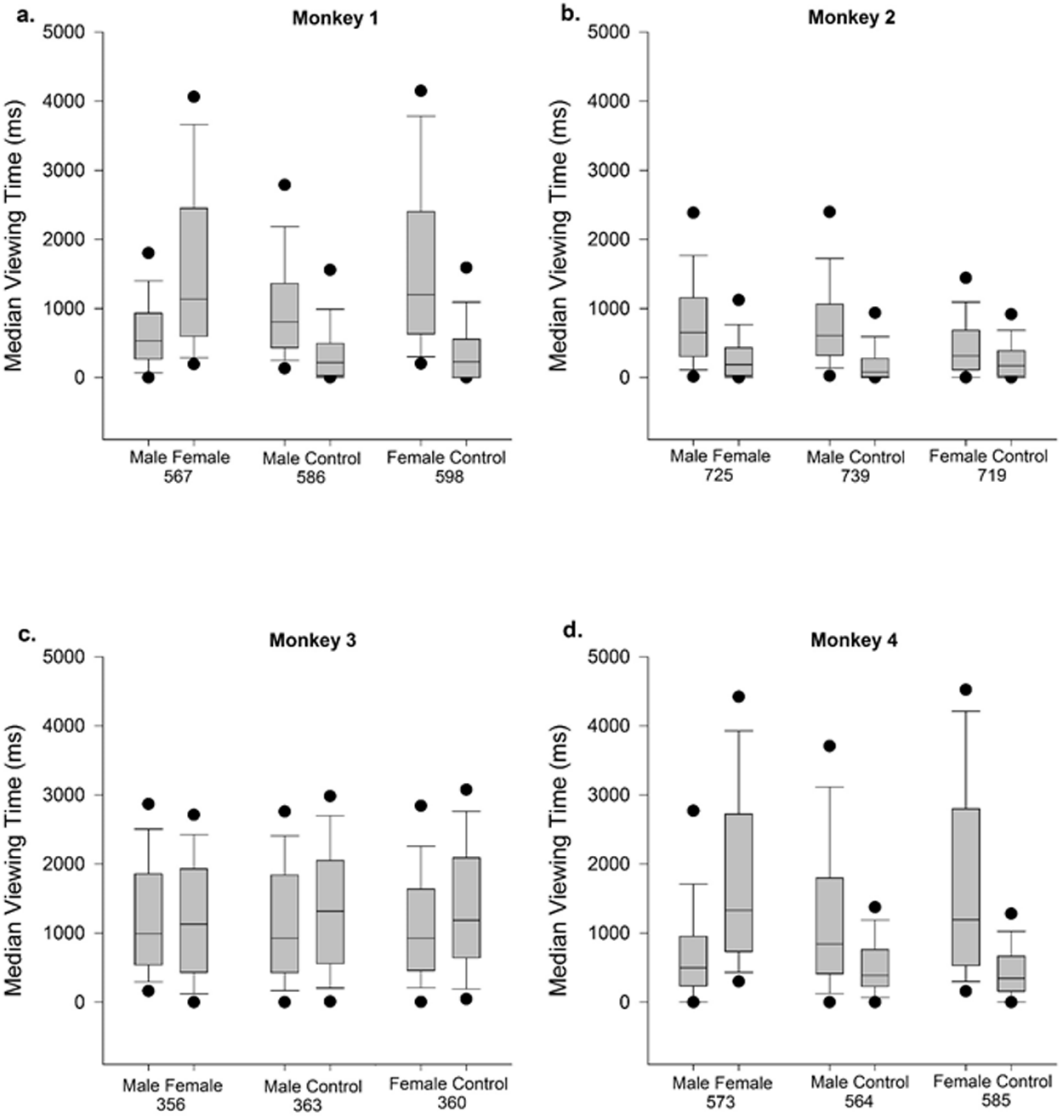
Median viewing times for pairs of images presented in the image preference test. a) Monkey 1, b) Monkey 2, c) Monkey 3, and d) Monkey 4. Closed circles represent the 5^th^ and 95^th^ percentiles. Numbers on the x-axis refer to the total number of each type of image pairing the monkey was presented with during the preference testing.

All four monkeys significantly preferred one type of image more than the other two, however, they varied in their preferences (Fig 4). Monkeys 1 and 4 spent longer looking at the female perinea than the male faces (Monkey 1: W = 11.70; Monkey 4: W = 13.19, both *p* < 0.001) or the scrambled control images (Monkey 1: W = 16.12; Monkey 4: W = 18.09, both *p* < 0.001). In addition, they viewed male faces for longer than scrambled controls (Monkey 1: W = 13.95; Monkey 4: W = 11.43, both *p* < 0.001). Monkey 2 preferred the male faces to the female perinea (W = 15.28, *p* < 0.001) and to the controls (W = 17.70, *p* < 0.001), moreover, he looked for longer at the female perinea than their scrambled controls (W = 8.70, *p* < 0.001). Surprisingly, Monkey 3 did not show a preference for either type of social stimulus, and instead looked at the scrambled control images for longer than the female perinea (W = 2.83, *p* = 0.005) and the male faces (W = 2.45, *p* = 0.014), and he had no preference between the male and female images (W = 0.04, *p* > 0.05). Therefore, in the fluid + image reward tasks, Monkeys 1 and 4 were shown images of female perinea, Monkey 2 was shown images of male faces, and Monkey 3 was shown scrambled control images.

### Using the preferred images as rewards

**Fig 5:**
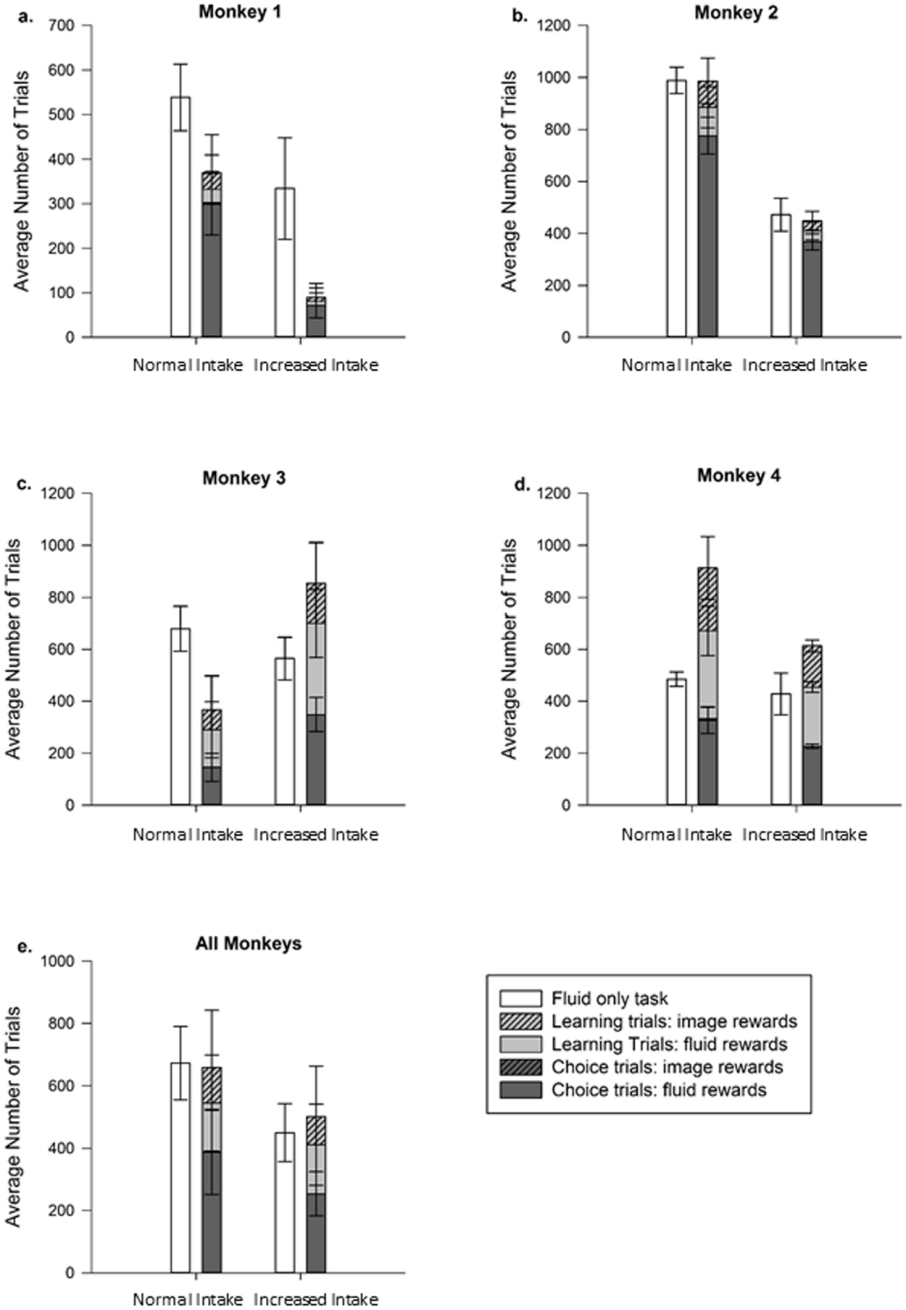
Average numbers of trials (±SEM) completed for the fluid only task (white bars) and the fluid + image reward task (all grey bar summed). Striped bars represent image rewards and solid bars represent fluid rewards. It should be noted that choices to view images were so rare that the bars are not visible in the figure. a) Monkey 1, b) Monkey 2, c) Monkey 3, d) Monkey 4 and e) all monkeys pooled.

At the normal fluid allowance, the average number of trials completed in the fluid + image reward task vs the fluid only task did not differ when data were pooled for all monkeys (χ^2^ (1) = 0.01, P > 0.05). When assessed individually, three of the monkeys exhibited the same effect (Monkey 1: *t*_(6)_ = 1.49; Monkey 2: *t*_(4.8)_ = 0.02; Monkey 3: *t*_(6)_ = 1.99, all *p* > 0.05; Fig 5a–5c). However, Monkey 4 (rewarded with colour images) performed more trials for the fluid + image reward task than for the fluid only task (Mean difference = 428, *t*_(6)_ = 3.00, *p* = 0.024; Fig 5d), suggesting that the inclusion of his preferred social stimuli increased his motivation to work. However, when assessing only the trials in which the monkeys had a choice between accessing a fluid or a preferred image reward, fluids were the favoured reward for all four monkeys, with all individuals choosing fluid rewards in over 98% of trials (Monkey 1 = 99.17% fluid choices, Monkeys 2 and 3 = 100%, Monkey 4 = 98.34%; Fig 5). These data show a strong preference for fluid rewards over preferred image rewards.

Given the strong preferences that all monkeys showed for fluid rewards at their normal fluid allowance, the daily fluid allowance was increased by 100 ml in an attempt to decrease motivation for fluids and assess whether this increased the motivational value of the preferred image rewards. 100 ml was an appropriate increase given that no monkey drank 100ml over his minimum volume on a daily basis across the 8 days (Median difference from minimum in ml: Monkey 1: -100; Monkey 2: 30; Monkey 3: -235; Monkey 4: -85). Increases of 100 ml correspond to the following percentage of FAI and ml/Kg/day: Monkey 1: 29% or 20 ml/Kg/day; Monkey 2: 74% or 35 ml/Kg/day; Monkey 3: 37% or 40 ml/Kg/day; Monkey 4: 61% or 34 ml/Kg/day. This increase in intake allowed an assessment of whether the social stimuli had any motivational value when the animals were potentially less motivated by fluids. The fluid + image reward task and the fluid only task were carried out for a further 8 days in the same order (ABBABAAB). To establish whether decreases in motivation to work for fluid rewards had occurred by increasing the daily fluid allowance by 100 ml, data from the fluid-only task were analysed. Data were normally distributed and *t* tests were used for each monkey to determine differences in task performance between the normal and increased fluid allowances. In addition, we performed the analysis for pooled data from all using a generalised estimating equation (GEE), adding in Monkey as the subject for repeated measures; we expected lower task performance once the fluid allowance was increased.

Despite the increased fluid allowance and apparent reductions in fluid intake during the study, motivation in the fluid-only task was only significantly decreased for Monkey 2 when assessed individually (Table 1). However, when data were pooled, monkeys performed significantly fewer trials after daily intake had been increased (GEE: χ^2^ (1) = 6.31, P = 0.012). During the increased fluid allowance, the average number of trials performed in the fluid only and the fluid + image reward tasks did not differ for any of the monkeys both when assessed together (χ^2^ = 0.02(1), P > 0.05) or individually (Monkey 1: *t*_(6)_ = 2.07; Monkey 2: *t*_(6)_ = 0.28; Monkey 3: *t*_(6)_ = 1.64; Monkey 4: *t*_(6)_ = 1.91, all *p* > 0.05), suggesting that working performance was still driven by fluids, and not by the image rewards (regardless of whether they were greyscale or colour). This finding is further strengthened by the fact that the increase in daily fluid allowance did not change the preference for fluid rewards, with all monkeys choosing fluid 100% of the time in choice trials.

**Table 1:** Difference in the number of trials performed for the control task at the normal fluid allowance and after fluid allowance had been raised by 100 ml and the associated *t* test values.

	Mean Difference	Standard Error of Difference	*t* value	df	*p* value
Monkey 1	-204.75	136.33	1.50	6	0.18
Monkey 2	-517.00	93.77	5.51	6	0.001
Monkey 3	-114.50	119.35	0.96	6	0.38
Monkey 4	-56.25	98.38	0.57	6	0.59

## Discussion

This study explored the social preferences of four laboratory rhesus macaques and the efficacy of using their preferred greyscale and colour stimuli as rewards for the successful completion of a single trial in a cognitive task. Despite earlier suggestions in the literature, we were unable to find evidence that social rewards could be used as an alternative (or supplement) to fluid rewards to motivate male macaques to participate for extended daily periods in a behavioural neuroscience task. These results have implications for refining current fluid restriction protocols for experimental animals.

Interestingly, the monkeys showed a wide range of preferences for the images shown to them. Whilst all four monkeys showed a strong preference for looking at one type of image, only three showed a preference for one of the social stimuli: two monkeys preferred to look at male faces, and one monkey favoured female perinea, as found in previous studies [20,21]. Surprisingly, one monkey preferred the scrambled control images over both female perinea and male faces. Speculatively, this could be because the control images were novel, abstract stimuli, the type of which he had not been previously exposed to. Taken together, the data clearly show that it cannot be assumed that all monkeys will be interested in looking at social stimuli, and when they do, individuals may not prefer the same type of social stimulus (see also [23]). This emphasises the need to ensure that if future attempts are made to use social stimuli as motivators in tasks, they need to be tailored to each individual prior to the experiment.

Given the clear and strong preferences to view one type of image that all the monkeys showed, it was also surprising not to see strong effects on animals’ motivation to access the images through performance in the tasks. At their normal fluid allowances, only one of the monkeys (Monkey 4) performed more trials for the fluid + image reward task than for the fluid-only task. The remaining three monkeys did not differ in performance between the two tasks. Although these data suggest that the use of images could be rewarding for some animals, and increase their task performance, it was evident that when given a choice between social and fluid rewards, fluids were favoured and chosen over images in more than 98% of the trials for each monkey. Therefore, even for Monkey 4, where the number of trials performed had increased with the inclusion of images, this was not driven by a motivation to voluntarily access social rewards, since they were rarely chosen. Consequently, there is no evidence that an animal’s motivation to work was driven for the desire to access social (or preferred) stimuli.

When the daily fluid allowance of the monkeys was increased in an attempt to decrease motivation for fluid rewards, the monkeys continued to show a strong preference for fluids over images when given the choice. This could be because the increase in daily fluid allowance was not sufficient to reduce motivation for fluid rewards. Consistent with this idea, a reduction in daily performance in the fluid-only task occurred for only one of the animals; however, even for this animal, there was no increase in viewing of social rewards with the increased daily allowance. The allowance could have been further increased for the three other animals, however, this would have unnecessarily increased the study length. This risked the monkeys becoming more familiar with the images, making the data difficult to interpret as changes in the monkeys’ motivation could have been confounded by habituation to the images.

Greyscale and colour images were not treated differently by the animals: individuals showed the same strong (individual) preferences for both types of social images. It was expected that the colour images would be more rewarding than the greyscale due the social salience of the red colouration. However, Waitt et al. [29] and Higham et al. [30] suggest that the signals may be more valuable to female macaques and in this study at least, there was no evidence that red coloration produced a stronger preference or was more rewarding for the animals (see also [20]).

Although there was some indication of motivation to view the images, the majority of the findings presented here are not in line with previous studies which have successfully reinforced monkeys with social rewards [20–22,31]. This could be due to the monkeys’ individual preferences for nutritive rewards instead of social rewards. This type of individual preference has been demonstrated previously in both rhesus macaques [32,33] and bonnet macaques (*Macaca radiata*) [22,34], with some animals having been shown to favour video rewards of conspecifics, others preferring to receive a food pellet reward, and some showing no definite preference for either. Therefore, although the monkeys in this study show preference for nutritive fluid rewards over social rewards, we cannot be certain that social rewards would not be rewarding in a different population of laboratory animals.

One possible reason for why a preference for a certain stimulus did not translate into motivating animals to perform a task is that the animals were socially housed. Every monkey was pair-housed, and had visual, auditory and olfactory contact with approximately 40 other rhesus macaques (both male and female) housed in the primate unit. Although some studies have successfully implemented social rewards with pair- and group-housed macaques (e.g. [20,21]), it may be that the level of social enrichment experienced on a daily basis by the monkeys in the study was too high for the images to be adequately valued as reward. It could be that images presented in cognitive tasks may be more rewarding to macaques that are socially restricted or deprived, such as those that are singly housed. However, we would emphasise that any reason for single housing animals has to be entirely justifiable, and do not consider that single housing animals for the purpose of rewarding them with social images a refinement to current protocols, especially in light of the limited impact fluid control has physiologically or behaviourally [7].

In this study, free-viewing image preferences established after the receipt of a valuable reward (fluid), did not translate into motivating rewards when paired against the choice of fluid rewards. This was potentially influenced by the many hundreds of times the images were presented in the preference testing stage of the experiment. This initial exposure to the images may have been sufficient for the monkeys to become habituated to, and uninterested by the stimuli; although previous studies have found evidence of prolonged exposure without habituation [34].

Finally, it is perhaps most likely that both the greyscale and colour stimuli are not sufficiently motivating on a trial-by-trial basis when many iterations of a task must be performed. The task used in the current study was very simple and motivation to perform a more complex task would need to be very high. Given the low level of motivation elicited by preferred images to perform a simple task, one may speculate that performance for social rewards would be poorer still for a more cognitively challenging task, though this is yet to be quantitatively confirmed. Perhaps, instead of using the images trial-by-trial, the stimuli would be more useful as a “jackpot” reward [6], occurring every 50 to 100 trials in addition to the fluid rewards, in order to enhance motivation, without risking an overuse the images. Alternatively, the stimuli could be used alongside smaller fluid rewards, as described by Deaner et al [20] in order to lessen the fluid restriction. A larger stimulus set containing more individuals may evoke a maintained interest in the images and changing the stimulus set has also been shown as an effective way to increase interest in social rewards [22]. However, the time and resources required to build a stimulus set such as that used in this study are not trivial. Moreover, implementing preferred stimuli presentation as rewards in an experimental setting where neuroscientific data are obtained is equally non trivial, as reward choices (preferred image vs. fluid) at the end of a trial increases the effort and trial time, which may thus offset the added motivation.

## Conclusions

In conclusion, we found that preferred social stimuli, whether greyscale or colour, were not sufficient for motivating rhesus macaques to perform trials in cognitive tasks that are regularly used in behavioural neuroscience. Based on these data, social rewards cannot be recommended as a viable strategy to refine fluid restriction protocols, although future studies that build on these findings may find alternative reward schedules that could theoretically overcome the currently encountered limitations.
